# Hepatorenal protective effects of essential oils against chemical overexposure induced oxidative damage

**DOI:** 10.3389/fphar.2025.1580805

**Published:** 2025-04-16

**Authors:** Faming Jiang, Xiaoyan Huang, Xingying Chen, Yuanhua Xian

**Affiliations:** ^1^ Faculty of Modern Agriculture, Yibin Vocational and Technical College, Yibin, China; ^2^ Engineering Center of Agricultural Biosafety Assessment and Biotechnology, School of Advanced Agricultural Sciences, Yibin Vocational and Technical College, Yibin, China; ^3^ Department of Traditional Chinese veterinary Medicine Assessment, Engineering Center of Agricultural Biosafety Assessment and Biotechnology, Yibin Vocational and Technical College, Yibin, China

**Keywords:** essential oil, oxidative stress, chemical overexposure, hepatorenal protective, medicinal potential

## Abstract

Chemical overexposure is a significant concern in both environmental and occupational settings, often leading to oxidative stress and subsequent cellular damage. The review covers the oxidative stress induced by chemical overexposure to substances such as pesticides (including pyrethroid, deltamethrin, β-cyfluthrin, malathion, triflumuron, methomyl, diquat, cypermethrin, thiamethoxam, and profenofos), medications (acetaminophen), nanoparticles (including zinc oxide, iron, silver, and titanium dioxide), heavy metals (including cadmium, vanadium), and some organic chemicals (including diethylnitrosamine and benzo(a)pyrene). Focusing on preclinical evidence from animal models and cell-based studies, essential oils have been shown to significantly enhance antioxidative enzyme activities, including superoxide dismutase, catalase, and glutathione peroxidase, while also increasing levels of non-enzymatic antioxidants such as glutathione and uric acid. Additionally, essential oils contribute to the restoration of biochemical parameters, including creatinine, uric acid, and urea levels, while mitigating oxidative damage by reducing cell membrane injury, apoptosis, and histopathological alterations. Additionally, this review evaluates the protective benefits of essential oils against chemical overexposure in animal models. The underlying mechanism is involved in alleviating hepatorenal damage. This review underscores the considerable promise of essential oils as candidates for medicinal applications in functional foods or medicines, particularly in preventing oxidative stress induced by various chemical overexposure.

## Graphical Abstract

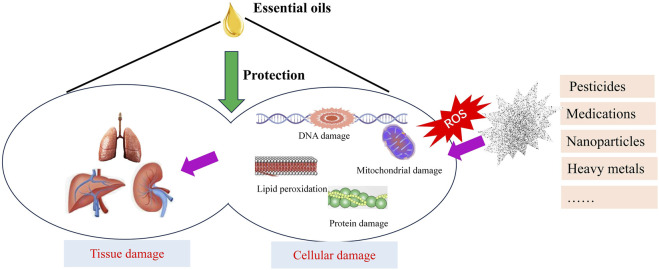

## 1 Introduction

Overexposure to pesticides, medicines, environmental pollutants, UV radiation, cigarette smoke, and other various chemicals are significant external sources of reactive oxygen species (ROS) (Sule et al., 2022). When ROS production overwhelms the body’s antioxidant defenses, it leads to cellular and molecular damage, affecting lipids, proteins, and DNA, and potentially disrupting normal cellular functions (Tian et al., 2025), contributing to chronic diseases such as cancer, cardiovascular disease, neurodegenerative disorders, and metabolic syndromes (Tiwana et al., 2024). For instance, occupational exposure to benzene and polycyclic aromatic hydrocarbons has been associated with elevated risks of leukemia and respiratory diseases (Chiavarini et al., 2024). Additionally, high levels of heavy metals like cadmium and lead have been correlated with kidney dysfunction and cognitive decline (Howard et al., 2024; Yang et al., 2025). These findings underscore the urgent need for effective antioxidant strategies to mitigate oxidative damage induced by chemical exposure.

Essential oils (EOs), as secondary metabolites, belong to the group of biologically active volatile organic compounds (Ivanova et al., 2025). They have been widely used in various fields including medicine, agriculture, cosmetics, perfumes, and as food condiment (He et al., 2023; Agnihotry et al., 2024). EOs offer health benefits to prevent infectious and chronic diseases, thus improving digestion, appetite, as well as regulating gut microbiota (Aziz et al., 2024). There is an increasing interest in utilizing EOs due to their bioherbicide potential (Poveda et al., 2025), antibacterial efficacy (Khwaza and Aderibigbe, 2025), and insecticidal properties (Li et al., 2025). In the food industry, studies have found that adding EOs from medicinal and aromatic plants can introduce various bioactive compounds exhibiting a range of medicinal potentials such as antiviral (Misra et al., 2024), antifungal (Zhou et al., 2025), antibiofilm (Devi et al., 2024), antibacterial activities (Khwaza and Aderibigbe, 2025), and anti-inflammatory effect (Huang et al., 2025), which help reduce fat oxidation and improve both food shelf-life and aromatic flavor (Liñán-Atero et al., 2024). Furthermore, many commodities using the essential oils as raw material are widely employed in aromatherapy, where they could relieve some psychological and/or physical disorders (Murugesh et al., 2024), such as anxiety and depression (Chavda et al., 2025).

EOs derived from plants have shown considerable medicinal potential as antioxidants in various conditions that involve oxidative stress. Their diverse bioactive compounds exhibit strong antioxidant properties through multiple mechanisms, including free radical scavenging (Messaoudi et al., 2024), metal ion chelation (Mohammed et al., 2024), and modulation of endogenous antioxidant systems (Kashyap, 2024). Compared to synthetic antioxidants or single-compound natural antioxidants, EOs offer a complex mixture of phytochemicals that may exert synergistic effects. EOs have been widely used in traditional medicine and functional foods, making them promising candidates for mitigating oxidative stress-related damage induced by chemical overexposure. Additionally, EOs derived from plants are natural, environmentally friendly, and sustainable candidates, offering a renewable resource with potential applications in health and wellness.

The aim of this review is to summarize the antioxidant potential of essential oils and their potential therapeutic effects on cellular oxidative damage induced by chemical overexposure. Additionally, this review analyzes the antioxidant mechanisms reported in different studies, highlighting the multifaceted actions of essential oils, which involve various bioactive compounds that scavenge free radicals, chelate metal ions, activate endogenous antioxidant systems, and regulate inflammation and autophagy. This review highlights their potential applications in functional foods and medicinal formulations for preventing oxidative stress.

## 2 Hepatorenal protection against pesticides overexposure

Overexposure to pesticides presents an ongoing health risk. It will lead to severe impairments to spleen, liver, kidney, and brain (Hussain et al., 2025). Overexposure could cause cognitive and neurological damage by modulating the permeability of voltage-gated sodium channels in nerve cells, causing repetitive neural impulses (Saravi and Dehpour, 2016; Erdman, 2016). In addition, pesticides exhibit more neurobiological affects, impacting cholinergic neurotransmission, dopaminergic, noradrenergic, and central *γ*-aminobutyric acid (GABA) (Huang et al., 2024; Menzikov et al., 2024). EOs extracted from plants have shown their antioxidative effects on tissues damage caused by pesticides overexposure (Table 1).

**TABLE 1:** The source of essential oils and their antioxidative effects on tissues damage caused by pesticides overexposure.

Plants	Experiment model	The dose/concentration of administration	Main action	References
*Origanum majorana*	Pyrethroid-induced liver injury in rats	160 mL/kg b.w. orally daily for 28 days	Hepato- protective effect	Mossa et al. (2013)
*Allium sativum*	Deltamethrin intoxicated rats	200 mg/kg b.w. orally daily for 4 weeks	Mending the hepatic toxicity	Ncir et al. (2020)
*Artemisia. campestris*	Deltamethrin intoxicated	200 mg/kg b.w daily for 2 weeks	Normalizing the altered serum levels of creatinin, urea, uric acid, and AChE	Saoudi et al. (2017)
*Ocimum basilicum*	β-cyfluthrin intoxicated rats	3 mL/kg, orally for 1 month daily	Hepato- protective effect	Jebur et al. (2022)
*Origanum vulgare*	Cypermethrin-Overexposed African catfish	0.5% and 1.0% dietary supplemented for 30 days	Modulating hepatorenal damage	Khafaga et al. (2020)
*Lavandula stoechas*	Malathion intoxicated mice	10, 30, and 50 mg/kg b.w. orally daily for 30 days	Mitigating hepatic and renal oxidative stress	Selmi et al. (2015)
*Pelargonium graveolens* (Geranium)	PFF-exposed carp, *Cyprinus carpio* (L.)	400 mg/kg dietary supplemented for 60days	Hepato- and nephron-protective effects	Rahman et al. (2020)
*Pelargonium graveolens*	Methomyl intoxicated rats	75 mg/kg b.w. orally for 28 days	Protecting the hippocampus	Amine et al. (2020)
*Thymus vulgaris*	TMX-exposed African catfish	500 ppm dietary supplemented for 1 month	Inhibiting hepatorenal damage, immunotoxicity and oxidative stress	El Euony et al. (2020)
*Faeniculum vulgare* Mill., (fennel)	TFM treated HCT116	Pretreated by FEO with 1, 1.5, and 2% (v/v) for 2 h	Decreasing DNA damages and mitochondrial membrane potential loss	Timoumi et al. (2020)
*Origanum vulgare L.* (Oregano)	Diquat treated rats	5 and 20 mg/kg b.w. orally for 14 days	Maintaining jejunal architecture	Wei et al. (2015)

Notes: b. w., body weight; AChE, acetylcholinesterase; HCT116, human carcinoma cells; TFM, triflumuron; TMX, thiamethoxam; PFF, profenofos.

### 2.1 Liver protection against pyrethroid insecticide

*Origanum majorana* essential oil demonstrated significant protection by normalizing marker enzymes, as well as replenishing of antioxidant levels, indicating the benefits in hepatic oxidative damage rats caused by pyrethroid (Mossa et al., 2013). Deltamethrin, an artificial pyrethroid possessing strong insecticidal properties has been shown to induce noteworthy elevated biochemical parameters including hepatic lipid peroxidation (LP), alanine aminotransferase (ALT), alkaline phosphatase (ALP), aspartate aminotransferase (AST), carbonyl protein, and advanced oxidized protein products (AOPP), along with a decreased glutathione (GSH), glutathione peroxidase (GPX), catalase (CAT) and SOD levels, which were validated by histological studies (Ncir et al., 2020). However, *Allium sativum* EO notably alleviated the hepatotoxicity in deltamethrin-treated rats (Ncir et al., 2020). *Artemisia. campestris* EO also showed its capability to normalize the modified acetylcholinesterase (AChE), acid, uric, urea and creatinine concentrations, and to reduce LP in deltamethrin-intoxicated rats (Saoudi et al., 2017).

Similarly, β-cyfluthrin, another pyrethroid insecticide, caused severe increase in LP parameters (H_2_O_2_ and thiobarbituric acid reactive substances (TBARS)), protein oxidation (hydroxyproline (HYP), AOPP, and protein carbonyl (PC)), proinflammatory gene expressions (interleukin (IL)-6, tumor necrosis factor-α (TNF-α)), cell cycle arrest in the G0/G1 phase of hepatocyte, a reduced cell number in G2/M and S phase, and DNA damage (Jebur et al., 2022). *Ocimum basilicum* essential oil could effectively abolish these adverse effects, restoring enzymatic (glutathione S-transferase (GST), glutathione reductase (GR), CAT, GPx, and superoxide dismutase (SOD)) and non-enzymatic antioxidants (such as GSH), as well as enzyme activities (lactate dehydrogenase (LDH), ALP, ALT, and AST), weights of liver and body, hematological markers, total bilirubin levels, globulin, and albumin (Jebur et al., 2022). This indicates that *O. basilicum* essential oil has a beneficial effect on liver protection.

Cypermethrin (CP) is extensively utilized in agriculture to protect crops against insects especially in cotton fields (Farhan et al., 2024). Essential oil from *Origanum vulgare* (OVEO) rich in phytochemicals like *p*-cymene, γ -terpinene, carvacrol, and thymol, possesses significant antioxidative capacity (Alagawany et al., 2020; Oniga et al., 2018). Dietary supplemented with OVEO resulted in a notable reduction in creatinine, urea, ALP, AST, and ALT levels (Khafaga et al., 2020). Apoptosis and histopathologic changes were notably diminished, accompanied by a simultaneous reduction of DNA damage in *Cyprinus carpio* overexposed to CP (Khafaga et al., 2020).

### 2.2 Hepatorenal protection against organophosphate insecticides

Besides, many research has found that EOs possess benefits in protecting against diverse insecticide overexposure. *Lavandula stoechas* essential oils (LSEO) has been shown to mitigate hepatic and renal oxidative stress induced by malathion including an increase in the levels of malondialdehyde (MDA) and H_2_O_2_, a reduction in antioxidants including total SOD, Cu/Zn-SOD, Mn-SOD, Fe-SOD, GPx, CAT, and sulfhydril (-SH) group level in the kidney and liver, suggesting potential nephro- and hepatoprotective effects in mice (Selmi et al., 2015).

Profenofos (PFF), one of organophosphate insecticides widely utilized to control various crop insects such as cotton, paddy, and tobacco (Majumder et al., 2024). Supplementation with Geranium essential oil (GEO) improved PFF-induced increases in ALT, AST, ALP, MDA, triglycerides (TG), cholesterol, urea, creatinine, and LDH levels (Rahman et al., 2020). It also mitigated the decline in antioxidative enzyme activities and GSH, NO, complement3 (C3), lysozyme activity, glucose (Glu), globulins, and total protein (TP) (Rahman et al., 2020). Consequently, GEO improved the histological structure in kidney and liver in PFF-exposed fish.

### 2.3 Hepatorenal protection against carbamate insecticides

*Pelargonium graveolens* Essential Oil (EO) have been found the protective effect on the hippocampus of methomyl-intoxicated rats, including histopathological alterations, as well as mended memory impairments due to its potential antioxidant actions (Amine et al., 2020).

### 2.4 Hepatorenal protection against neonicotinoid insecticides

In aquaculture, EOs have also shown protective effects against various insecticide overexposure. Similarly, the concurrent addition of *Thymus vulgaris* essential oil to the diet of African catfish (*Clarias garipenus*) alleviated histomorphometric and histopathological damage due to thiamethoxam (TMX) toxication, one of neonicotinoid insecticides widely used for controlling cotton pests and various crop pests (El Euony et al., 2020). It modulated immunotoxicity, oxidative stress, and hepatorenal damage, and increased caspase-3 immunopositive splenocytes and proliferating cell nuclear antigen (PCNA) levels (El Euony et al., 2020).

### 2.5 Hepatorenal protection against insect growth regulators

Similarly, fennel essential oil (FEO) pretreatment for 2 h resulted in modulation in CAT and SOD activities, reduction in the mitochondrial membrane potential loss DNA damage, MDA levels, and ROS production, thus elevating cell viability in human carcinoma cells (HCT116) subjected to oxidative stress induced by triflumuron (TFM) (Timoumi et al., 2020).

### 2.6 Hepatorenal protection against herbicides

In an experiment, Oregano (*O. vulgare L.*) EO could counteract oxidative damage in jejunum of diquat-intoxicated rats, thus preserving its structure (Wei et al., 2015). The underlying mechanism may be associated with modulating specific enteric microorganism, inhibiting inflammatory cytokines level, consequent elevating occludin content (Wei et al., 2015).

Thus, EOs are promising candidates due to their benefits performed in pesticide overexposure. They have shown excellent ameliorative effects on toxic impacts related to oxidative stress, immunological markers, and hepato-renal functions.

## 3 Liver protection against overdoses medications

Although effective medications with therapeutic doses are commonly hold in households, administering them in an overdose can lead to grievous acute hepatic failure, cell death, hepatic injury, and even high mortality within both animal and human (Greydanus et al., 2023). Paracetamol (PCM), also known as acetaminophen (N-acetyl-p-aminophenol, APAP), is one of the most widely used analgesics. APAP overdose will lead to hepatic damage, leading to acute liver failure (Cardia et al., 2021; Dadkhah et al., 2015). Its toxic effect occurs when taken in a single or repeated high dose or after chronic ingestion (Dear, 2024). Herbal EOs have shown their antioxidative effects on tissues damage caused by overdoses medications (Table 2).

**TABLE 2:** Essential oils plant name and their antioxidative effects on tissues damage caused by overdoses medications and nanoparticles.

Plants	Experiment model	The dose/concentration of administration	Main action	References
Overdoses medications				
*Lavandula officinalis*	APAP-intoxicated mice	200 and 400 mg/kg, once daily for 7 days	Inhibiting hepatorenal damage, immunotoxicity and oxidative stress	Cardia et al. (2021)
*Achillea wilhelmsii* C. Koch (Asteraceae)	APAP-intoxicated rats	100 and 200 mg/kg, i.p. i one time after APAP administration	Reversal of hepatic necrosis	Dadkhah et al. (2015)
*Elettaria cardamomum* (cardamom)	PCM-intoxicated rats	100 mg/kg b.w. for 2 weeks	Protecting liver and kidney	Khattab et al. (2020)
*Salvia officinalis L.* (sage)	Co-amoxiclav overdose rats	30 mg/kg orally for 7 days	Inducing liver damage	El-Hosseiny et al. (2016)
*Hypericum scabrum*	APAP-intoxicated rats	100 and 200 mg/kg b.w, i.p one time after APAP administration	Inhibiting hepatorenal damage	Dadkhah et al. (2014)
*Schisandra chinensis* (Turcz.) Baill. (Magnoliaceae)	APAP overdose mice	0.25, 0.5, 1, 2 g/kg, gavaged once daily for 7 days	Mitigating liver injury	Zhao et al. (2022)
Nanoparticles				
*Thymus vulgaris* L. (thyme)	ZnO-NPs toxicity in rats	50, 100 mg/kg b.w. orally for 3 weeks	Alleviating histological changes, ctyogentical, biochemicaland, DNA damage, oxidative stress	Hassan et al. (2021)
*Ocimum basilicum* L (basil)	Fe-NPs toxicity in rats	100, 200 mg/kg b.w. orally for 28 days	Protecting liver and kidney	El-Nekeety et al. (2021)
*Chenopodium murale*	AgNPs toxicity in rats	0.5 or 1 mg/kg b.w orally for 21 days	Protecting kidney	Abdel-Wahhab et al. (2020)
*Thymus vulgaris* L. (thyme)	TiO_2_NPs toxicity in rats	50, 100 mg/kg b.w orally for 30 days	Protecting liver and kidney, Eliminating genotoxicity	Abdel-Wahhab et al. (2021)
*Thymus vulgaris* L. (thyme)	TiO_2_NPs toxicity in rats	5 mg/kg b.w orally for 21 days	Inhibiting disturbances in gene expression and DNA damage	Sallam et al. (2022)

Notes: APAP, acetaminophen (N-acetyl-p-aminophenol); b. w., body weight; i. p., intraperitoneal; PCM, paracetamol; ZnO-NPs, zinc oxide nanoparticles; Fe-NPs, iron nanoparticles; AgNPs, silver nanoparticles; TiO_2_NPs, titanium dioxide nanoparticles.

Studies on paracetamol-intoxicated rats have revealed significant decreases in total protein and albumin, along with marked hyperglycemia and elevation in serum transaminases, urea and creatinine (Khattab et al., 2020). These toxic effects were significantly ameliorated in groups receiving cardamom oil or silymarin (Khattab et al., 2020). Administration of cardamom essential oil significantly improved hepato-renal profiles, elevated total antioxidative activity, and protected livers and kidneys from histopathological alterations within PCM-intoxicated rats due to its potent antioxidant effect (Khattab et al., 2020). Additionally, essential oil from *Salvia officinalis L.* (sage) alleviated liver damages in Co-amoxiclav-intoxicated rat models by enhancing antioxidant enzymes levels, limiting LP and hence reducing cell membrane injuries (El-Hosseiny et al., 2016).

The protective role of *Lavandula officinalis* essential oil on APAP-intoxicated hepatic injury in mice demonstrated a decrease within gamma-glutamyl transferase (γ-GT), ALP, AST, and ALT contents, as well as reductions within pro-inflammatory cytokines, NO, and myeloperoxidase (MPO) contents (Cardia et al., 2021). Furthermore, pre-treatment with *L. officinalis* essential oil demonstrated notable antioxidative activity through reducing ROS, carbonylated proteins, and MDA contents, while enhancing oxidized GSH, CAT, and SOD contents in the liver (Cardia et al., 2021). This suggests that this essential oil improves hepatic function via suppressing inflammatory responses and oxidative stress (Cardia et al., 2021). In rats with APAP-induced liver damages, *Hypericum scabrum* essential oils reversed enhanced LP contents in the liver, along with the ferric reducing ability of plasma (FRAP), and restored depleted GSH levels, as confirmed by the histological examination of liver biopsies (Dadkhah et al., 2014). Similar to *H. scabrum* essential oils, *Achillea wilhelmsii* C. Koch (Asteraceae) EOs significantly mitigated increased contents of FRAP and LP, compensated SOD and GSH levels, as indicated by histological examination showing reversal of hepatic necrosis (Dadkhah et al., 2015). In another APAP-overdosed mice, pretreatment with *Schisandra chinensis* (Turcz.) Baill. (Magnoliaceae) (SC) EO reduced IL-6, TNF-a, ALT, and AST contents in serum, mended GSH depletion and MDA accumulation, and inhibited cytochrome P450 2E1 (CYP2E1) in the liver (Zhao et al., 2022). This mitigation of liver injury is linked to increased expression of nuclear factor erythroid 2 (NFE2)-related factor 2 (Nrf2), glutamate-cysteine ligase (GCL), and heme oxygenase-1 (HO-1), along with the activation of autophagy through upregulation of hepatic light chain 3 (LC3)-Ⅱ and decreased p62 (Zhao et al., 2022).

These findings propose that EOs mediate their hepatoprotective effect through activation of antioxidant defense and/or autophagy, suggesting their potential use in the treatment of medications overdoses.

## 4 Hepatorenal protection against nanoparticles

In recent years, nanoparticles (NPs) have been utilized for delivering nucleic acids, polypeptides, vaccines, genes, proteins, and drugs because of the unique physicochemical characteristics (Souto et al., 2024; Tenchov et al., 2025; Yuan et al., 2025). In another hand, concerns regarding the toxicity and/or safety in nanoparticles are raising such as zinc oxide nanoparticles (ZnO-NPs), iron nanoparticles (Fe-NPs), silver nanoparticles (AgNPs) and titanium dioxide nanoparticles (TiO_2_NPs).

### 4.1 Hepatorenal protection against zinc oxide nanoparticles

Studies have shown that ZnO-NPs may induce histological changes, ctyogenetic abnormalities, biochemical alterations, DNA damage, and oxidative stress (Hassan et al., 2021). Thyme essential oil (TEO) has been found to alleviate these disturbances, showing protective effects against the hazards of ZnO-NPs (Hassan et al., 2021).

### 4.2 Hepatorenal protection against iron nanoparticles

Similarly, rats exhibited notable elevation in DNA damage, lipid profiles, oxidative stress indicators, biochemical markers, cytokines, and regulation in antioxidative enzymes expression in liver and kidney tissues induced by Fe-NPs (El-Nekeety et al., 2021). The applications of Fe-NPs combined with basil essential oil (BEO) was shown to alleviate these disturbances, with high doses normalizing most indicators and improving the histological architecture in kidney and liver tissues (El-Nekeety et al., 2021).

### 4.3 Kidney protection against silver nanoparticles

Administration with AgNPs might enhance a harmful effect on the environment and human health. AgNPs have been found to disrupt renal functions and histological architecture, increase NO, MDA, TNF-α and serum electrolytes levels, decrease the antioxidative enzymes, downregulate P53 and Bax, upregulate Bcl-2, thus inducing histomorphometric damage (Abdel-Wahhab et al., 2020). *Chenopodium murale* essential oil (CMEO) has also been shown to protect renal functions encountering silver nanoparticles in a dose-dependent manner (Abdel-Wahhab et al., 2020).

### 4.4 Hepatorenal protection against titanium dioxide nanoparticles

Recently, titanium dioxide nanoparticles (TiO_2_NPs) have been widely used in various industries. These nanoparticles can lead to cell apoptosis, DNA damage, inflammatory response, and oxidative stress (Li and Tang, 2024; Manzoor et al., 2024). Numerous studies have reported that the administration of TiO_2_NP causes significant disturbances in liver and kidney function, lipid profile, inflammatory cytokines, gene expressions, and antioxidant properties in the liver and kidney (Abdel-Wahhab et al., 2021; Sallam et al., 2022; Salman et al., 2021). Additionally, there is an increase in sperm abnormalities, DNA damage in hepatic cells, chromosomal aberrations in bone marrow and germ cells, and histomorphometric alteration in the testis, kidney, and liver (Abdel-Wahhab et al., 2021; Sallam et al., 2022; Salman et al., 2021). In contrast, thyme EO has been found to improve all these parameters (Abdel-Wahhab et al., 2021; Sallam et al., 2022). Moreover, the co-administration of TiO2NPs and cinnamon essential oil (CEO) has been shown to alleviate these disturbances, enhance antioxidant capacity, and protect against TiO_2_NPs-induced oxidative damage and genotoxicity in male mice (Salman et al., 2021).

Thus, caution is advised when utilizing synthesized metal nanoparticles in various applications, as they may induce hepatic and renal toxicity and oxidative damage. In this context, EOs appear to be promising and safe candidates for mitigating hepatotoxicity and nephrotoxicity, providing protection against the oxidative stress and health hazards associated with metal nanoparticles. This suggests that EOs can be effectively applied in both food and pharmaceutical industries.

## 5 Hepatorenal protection against environmental pollutants

Essential oils from plant exert their antioxidative effects on tissues damage caused by environmental contaminants (Table 3).

**TABLE 3:** Essential oils plant name and their antioxidative effects on tissues damage caused by environmental contaminants, and hepatoprotective.

Plants	Experiment model	The dose/concentration of administration	Main action	References
Heavy metals				
*Thymus serrulatus*	Cd-intoxicated rats	100 and 200 mg/kg for 7 days	Ameliorating nephrotoxicity	Ansari et al. (2021)
*Rosmarinus officinalis*	CrVI-intoxicated rats	0.5 mL/kg b.w. orally for 14 days	Improvement in kidney tissue architecture	El-Demerdash et al. (2021)
Organic chemicals				
*Salvia officinalis*	Vanadium-intoxicated rats	15 mg/kg b.w. for 4 or 10 days	Protect against oxidative damage in livers	Koubaa et al. (2021)
*Elettaria cardamomum* (cardamom)	DENA-intoxicated rats	100 and 200 mg/kg orally for 7 days	Improving antioxidative activity in the brain and kidneys	Elguindy et al. (2018)
Air contaminants and irradiation				
*Zingiber officinale R* (Ginger)	Mice exposed to 6Gy	100 and 500 mg/kg b.w. orally for 5 days	Protecting against cellular DNA damage in bone	Jeena et al. (2016)
*Cymbopogon citratus*, Stapf (Lemongrass)	HELF cells treated with BaP	concentrations: 0.5%, 1%, and 2.5% for 24 h	Improving antioxidative activity and reducing the loss in cell viability, DNA damage	Jiang et al. (2017)
Chemical-induced hepatic injury animal models				
*Heracleum persicum*	CCl4 treatment rat model	100 & 200 mg/kg b.w. i.p. one time	Hepato-protective effect	Roshanaei et al. (2017)
*Cymbopogon citratus* (Lemongrass)	CCl4 treatment rat model	0.1, 0.2, 0.3 mL/kg for 5 days	Against hepatic/renal damage and genotoxicity	Fahmy et al. (2020)
*Pimpinella diversifolia* DC.	LPS/D-GalN-induced acute liver injury mouse model	50, 150 mg/kg i.p. once a day for 3 days	Alleviating liver injury, anti-inflammatory activity	Hua et al. (2023)
*Allium sativum* L. (Garlic)	Obese mice with long-term HFD-induced NAFLD	25, 50, and 100 mg/kg for 12 weeks	Hepato-protective effect	Lai et al. (2014)

Notes: Cd, cadmium; CrVI, chromium hexavalent; DENA, diethylnitrosamine; BaP, Benzo(a)pyrene; HELF, human embryonic lung fibroblast; PM_2.5_, particulate matter 2.5 μm or less in diameter; 6Gy, gamma-irradiation; CCl4, carbon tetrachloride; LPS, lipopolysaccharide; D-GalN, D-galactosamine; HFD, high-fat diet; NAFLD, nonalcoholic fatty liver disease; b. w., body weight; i. p., intraperitoneal.

### 5.1 Kidney protection against heavy mentals

Overexposure to chemicals in environment continues to be significant global public health issues. Cadmium (Cd) overexposure, an ecologically dangerous toxic metal, has increased in the biosphere from both anthropogenic and natural sources (Eka Putri et al., 2025; Mohammed et al., 2025). Previous studies have reported that Cd toxicity leads to irreversible renal tubule dysfunction, impairing the removal of toxic chemicals and drugs, which can result in acute or chronic kidney failure, and if untreated, may lead to death (Lang and Schiffl, 2024; Satarug, 2024). *Thymus serrulatus* essential oil (TSAEO) could significantly improve elevated LP, non-enzymatic antioxidants, as well as kidney function biomarkers (Ansari et al., 2021). It also downregulated the increased expression of inducible nitric oxide synthase (iNOS), nuclear factor kappa-light-chain-enhancer of activated B cells (NF-κB) p65, and Smad2 while ameliorating alteration in renal tissue of Cd-overexposure rat models (Ansari et al., 2021). Similarly, chromium hexavalent (CrVI)-intoxicated rats exhibited increased oxidative damage profiles (H_2_O_2_, TBARS), kidney function parameters (uric acid (UA), creatinine, urea), along with significant declined enzymatic antioxidants (GST, GPx, CAT, and SOD), total protein level, and GSH, as well as altered histology of kidneys (El-Demerdash et al., 2021). Treatment with *Rosmarinus officinalis* essential oil (REO) before or after CrVI exposure observably mended most of the biomarkers and improved kidney tissue architecture (El-Demerdash et al., 2021). In another experiment, administration of vanadium (NH_4_VO_3_) in rats enhanced LDH, ALP, AST, and ALT activities, as well as NO, triglyceride, bilirubin, and cholesterol contents, while reducing antioxidant enzyme activities in the liver (Koubaa et al., 2021). However, this disturbance was markedly restored by application of *Salvia officinalis* (SO) EO, which also mitigated histopathological alteration and heat shock protein (HSP)72/73 overexpression, suggesting a protective effect of EO from *S. officinalis* on oxidative stress in liver of vanadium-intoxicated rats (Koubaa et al., 2021).

### 5.2 Kidney and brain protection against organic chemicals

Diethylnitrosamine (DENA) is a toxic organic compound and potent carcinogen present in air, water, and soil (Limbu and Dakshanamurthy, 2023). The EO of *Elettaria cardamomum* (cardamom) has been found to enhance antioxidative capacities (CAT, SOD, GPx, GR, GST) in DENA-intoxicated rats’ brains and kidneys (Elguindy et al., 2018). Additionally, administration of cardamom and geraniol, a primary component of cardamom essential oil, resulted in a reduction of oxidative stress markers such as LP and the activity of ornithine decarboxylase (ODC) in the brain and kidney (Elguindy et al., 2018). It also increased GSH level of brain and kidney, and decreased AChE capacity of brain (Elguindy et al., 2018). Benzo(a)pyrene (BaP), a well-known environmental pollutant, is typically produced from organic materials’ incomplete combustion such as notably cigarette smoke, fossil fuel, and automobile exhaust (Ezugwu et al., 2024). BaP could be metabolized within cells, and its metabolites may contribute to carcinogenic processes (Hu et al., 2025). The administration of lemongrass (*Cymbopogon citratus*, Stapf) essential oil (LEO) to human embryonic lung fibroblast (HELF) cells exposed to BaP has been shown to enhance SOD and CAT activities and reduce MDA levels (Jiang et al., 2017). Furthermore, this EO can mitigate DNA damages and reductions in cell viability, as indicated by decreased 8-hydroxy-deoxyguanosine (8-OHdG) content (Jiang et al., 2017).

### 5.3 Brain and lung protection against air contaminants

Particulate matter micrometers or less in diameter increases MDA, NF-κB, nicotinamide adenine dinucleotide phosphate (NADPH) oxidase 2 (Nox2) and ROS levels, whereas reducing levels of SOD and HO-1, resulting in brain’s oxidative damage (Amin et al., 2025; Kim et al., 2020). Mentha piperita essential oil could inhibit asthma subjected to PM10 by regulating the IL-6/JAK2/STAT3 signaling pathway (Kim et al., 2020). Compound essential oils (CEOs) showed protective benefits of alleviating PM_2.5_-induced autophagy and oxidative damage through inhibiting the 5′-adenosine monophosphate-activated protein kinase (AMPK)/mammalian target of rapamycin (mTOR) signaling pathway, offering a promising therapy in PM_2.5_-induced brain and lung damage (Ren et al., 2021).

The above results reveal that EOs have beneficial effects in ameliorating injuries in the brain, liver, kidney, lung, and bone marrow induced by toxic metals, organic compounds, and other environmental pollutants. This is attributed to their ability to modulate detoxification enzymes, enhance anti-oxidant stress capacities, and free radical scavenging. These results offer a novel opportunity for the prevention or treatment in environmental contaminant-induced damage.

## 6 Hepatoprotective mechanism

Various animal models are employed to observe protective benefits of essential oils for treating hepatic injury, aiming to identify potential hepatoprotective agents. Carbon tetrachloride (CCl4), commonly utilized for inducing experimental hepatic damage in laboratory, is a known environmental pollutant (Fareed et al., 2024). Essential oils from *Heracleum persicum* have been shown to modulate oxidative stress/antioxidant disturbance, reduce hepatic lipid peroxides, and regulate levels of GSH and GST concomitant, alongside adapting plasma FRAP, AST and ALT levels in CCl4-treated rat models (Roshanaei et al., 2017). *Cymbopogon citratus* essential oil (CCEO) has also demonstrated protective effects against hepatic and renal damage and genotoxicity, reversing the increase in oxidative damage parameters (MDA), creatinine, blood urea, and hepatic enzyme biomarkers (γ-GT, ALP, AST, ALT) in rats induced by CCl4 (Fahmy et al., 2020).

In other models, EO from *Pimpinella diversifolia* has been shown its hepatoprotective effect in acute hepatic damage mouse models using lipopolysaccharide (LPS) and D-galactosamine (D-GalN) (Hua et al., 2023). The findings revealed that administration of PDREO dramatically decreased serum ALT and AST levels, and effectively alleviated hemorrhage, edema, necrosis, and apoptosis in hepatic cells (Hua et al., 2023). This effect might result from the notable anti-inflammatory actions (limiting monocyte-derived neutrophils and macrophages infiltration, and reducing inflammatory chemokines and cytokines contents) and its regulation in mending oxidative damage (boosting antioxidative enzyme expressions including CAT, SOD1, GPX4) (Hua et al., 2023). Additionally, the EO at 150 mg/kg could entirely prevent LPS/D-GalN-induced mortality in mice (Hua et al., 2023). Administration of EO from garlic and diallyl disulfide (DADS) notably limited pro-inflammatory cytokine secretions in the liver, along with enhanced antioxidative ability that is inhibiting cytochrome P450 2E1 formation in obesity mice with nonalcoholic fatty liver disease (NAFLD) induced by long-term high-fat diet (HFD) (Lai et al., 2014). The actions might be mediated through downregulating 3-hydroxy-3-methylglutaryl-coenzyme A reductase, acetyl-CoA carboxylase, fatty acid synthase, and sterol regulatory element-binding protein-1c, accompanied by stimulating carnitine palmitoyltransferase-1and peroxisome proliferator-activated receptor α (Lai et al., 2014).

Ongoing external chemical substances exposed eventually results in numerous diseases. Xenobiotic toxicity primarily affects the liver (Rovira et al., 2024). Due to its capacity for concentrating xenobiotics and the predominant function in metabolism, the liver is more susceptible to damage from these chemical substances than any other organs (Rovira et al., 2024). EO components are rapidly absorbed through oral, pulmonary, or dermal routes (Al-Harrasi et al., 2022). After absorption, they are either metabolized or distributed into adipose tissues and organs (Sadgrove et al., 2021). At low doses, plasma levels return to baseline within 1–3 h (Sadgrove et al., 2021). However, at higher doses, plasma concentrations can be sustained for several days due to buffering from body tissues (Sadgrove et al., 2021). The distribution hierarchy is typically adipose tissue > liver > kidneys > cerebrospinal fluids and brain (Sadgrove et al., 2021). The solubility of EO constituents significantly influences their absorption in the gastrointestinal tract, where they may interact with digested food, potentially reducing solubilization and absorption (Horky et al., 2019). Once absorbed, most EOs undergo extensive metabolism, primarily in the liver (Dhifi et al., 2016). Some EOs, such as terpenes and phenolic compounds, can interact with hepatic cytochrome P450 enzymes, potentially affecting drug metabolism by either inhibiting or inducing enzymatic activity (Zehetner et al., 2019). This modulation can influence the metabolism and detoxification of xenobiotics, thereby affecting their bioactivation or clearance. A substantial portion of EO compounds is excreted via the kidneys, as indicated by increased urinary metabolites (Horky et al., 2019). EO compounds are generally characterized by rapid metabolism and short half-lives, minimizing the risk of accumulation in body tissues (Al-Harrasi et al., 2022).

**FIGURE 1:**
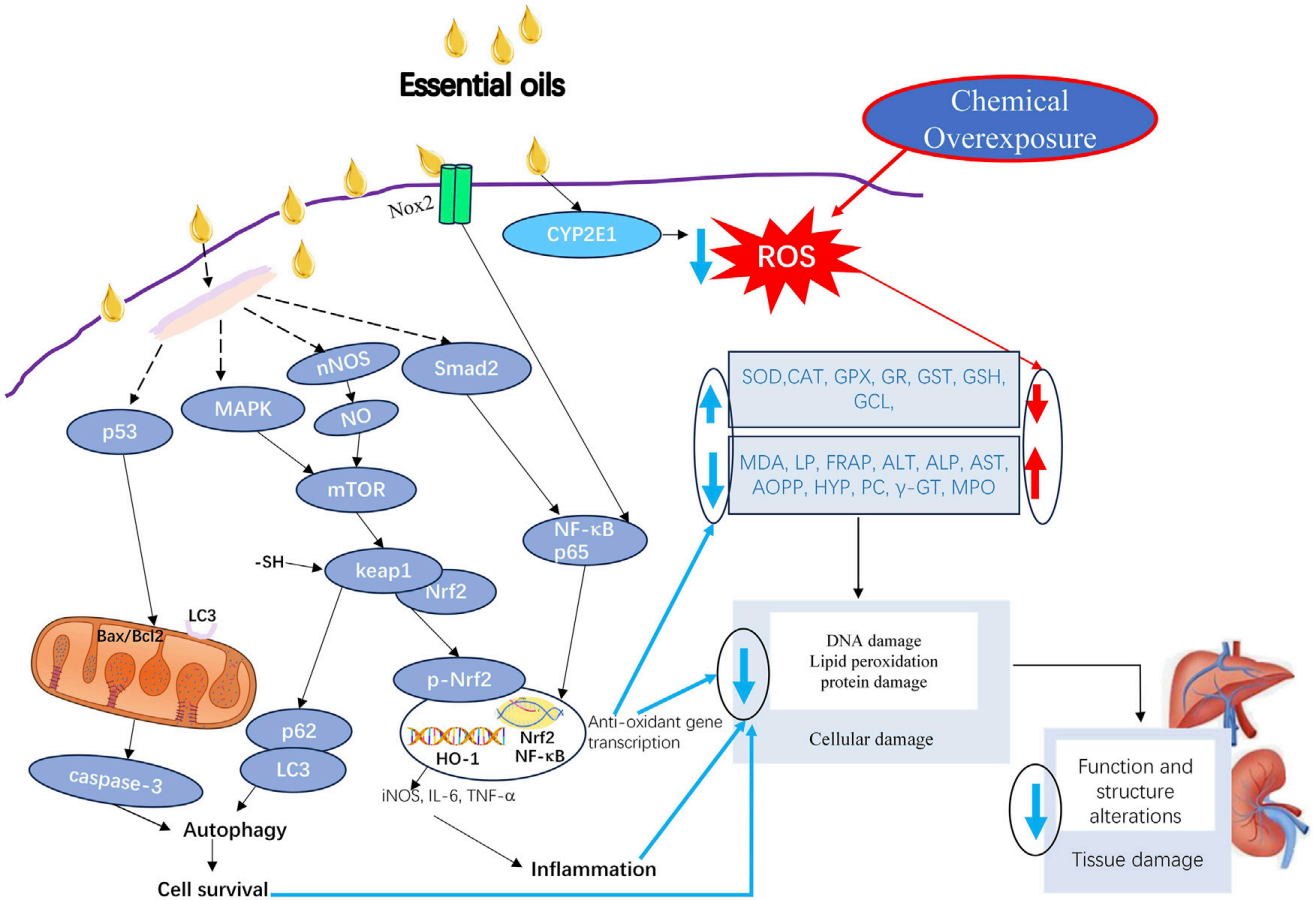
The underlying protective mechanism of EOs against oxidative damage induced by chemical overexposure. (1) Mitochondrial Protection: EOs suppress the expression of P53 and Bax, and upregulate Bcl-2 expression, thereby preventing mitochondrial dysfunction and reducing cellular damage. (2) Autophagy Modulation: Certain EOs activate autophagy via the p53 signaling pathway, leading to the upregulation of hepatic LC3-Ⅱ, which facilitates the clearance of necrotic cells. Conversely, other EOs inhibit autophagy through the AMPK/mTOR pathway and p62, promoting liver cell survival under stress conditions. (3) Antioxidant gene transcription: EOs enhance the expression of anti-oxidative genes such as HO-1 through activation of the Keap1/Nrf2 or Smad2/NF-κB p65 pathways, thereby leading to increase in SOD, CAT, GPX, GR, GST, GSH, GCL and decrease in MDA, LP, FRAP, ALT, ALP, AST, AOPP, HYP, PC, γ-GT, and MPO. (4) Anti-inflammatory Effects: EOs modulate signaling pathways to suppress the expression of IL-6 and TNF-α, thereby inhibiting inflammation. (5) Alleviating cellular damage: through the combined effects of the above factors, DNA damage, lipid peroxidation, and protein damage are reduced, ultimately alleviating cellular damage. (6) Alleviating tissue damage: All the mechanisms work together to mitigate tissue injury and maintain both its structural integrity and functional capacity. ALP, alkaline phosphatase; ALT, alanine aminotransferase; AOPP, advanced oxidized protein products; AST, aspartate aminotransferase; CAT, catalase; CYP2E1, cytochrome P450 2E1; FRAP, ferric reducing ability of plasma; GCL, glutamate-cysteine ligase; GPX, glutathione peroxidase; GR, glutathione reductase; GST, glutathione S-transferases; GSH, glutathione; HO-1, heme oxygenase-1; HYP, hydroxyproline; IL, interleukin; LC3, light chain 3; LP, lipid peroxidation; MAPK, mitogen-Activated Protein Kinase; MDA, malondialdehyde; MPO, myeloperoxidase; mTOR, mammalian target of rapamycin; NF-κB, nuclear factor kappa-light-chain-enhancer of activated B cells; Nox2, nicotinamide adenine dinucleotide phosphate (NADPH) oxidase 2; NOS, nitric oxide synthase; Nrf2, nuclear factor erythroid 2 (NFE2)-related factor 2; PC, protein carbonyl; ROS, reactive oxygen species; SOD, superoxide dismutase; TNF, tumor necrosis factor; γ-GT, gamma-glutamyl transferase.

While current research demonstrates the antioxidant potential and the underlying mechanism of EOs (Figure 1), many mechanisms remain unknown. Generally, EOs, once absorbed, undergo extensive metabolism in the liver, where they can modulate oxidative stress, influencing cellular survival and tissue integrity (Dhifi et al., 2016). One key mechanism involves the regulation of autophagy. Certain EOs activate autophagy via p53 signaling, leading to the upregulation of hepatic light chain 3 (LC3)-Ⅱ, which facilitates the clearance of necrotic cells (Zhao et al., 2022). Conversely, other EOs inhibit autophagy through the AMPK/mTOR pathway and p62, promoting liver cell survival under stress conditions (Zhao et al., 2022). EOs also exert antioxidant effects by enhancing the expression of anti-oxidative genes such as heme oxygenase-1 (HO-1). This occurs through the activation of the Keap1/Nrf2 or Smad2/NF-κB p65 pathways, which subsequently reduce oxidative stress and inhibit inflammatory responses (Hua et al., 2023; Zhao et al., 2022). Additionally, mitochondrial protection is achieved by the suppression of P53 and Bax, the upregulation of Bcl-2, preventing mitochondrial dysfunction and associated cellular damage (Huang et al., 2025). Moreover, some EOs have been shown to reduce chemical overexposure-induced fibrosis, thereby mitigating morphological damage in the liver (Fareed et al., 2024). Through the combined effects of the above factors, DNA damage, lipid peroxidation, and protein damage are reduced, ultimately alleviating cellular damage. This reduction in cellular damage helps minimize tissue injury and preserve both the function and structure of the tissue.

**FIGURE 2:**
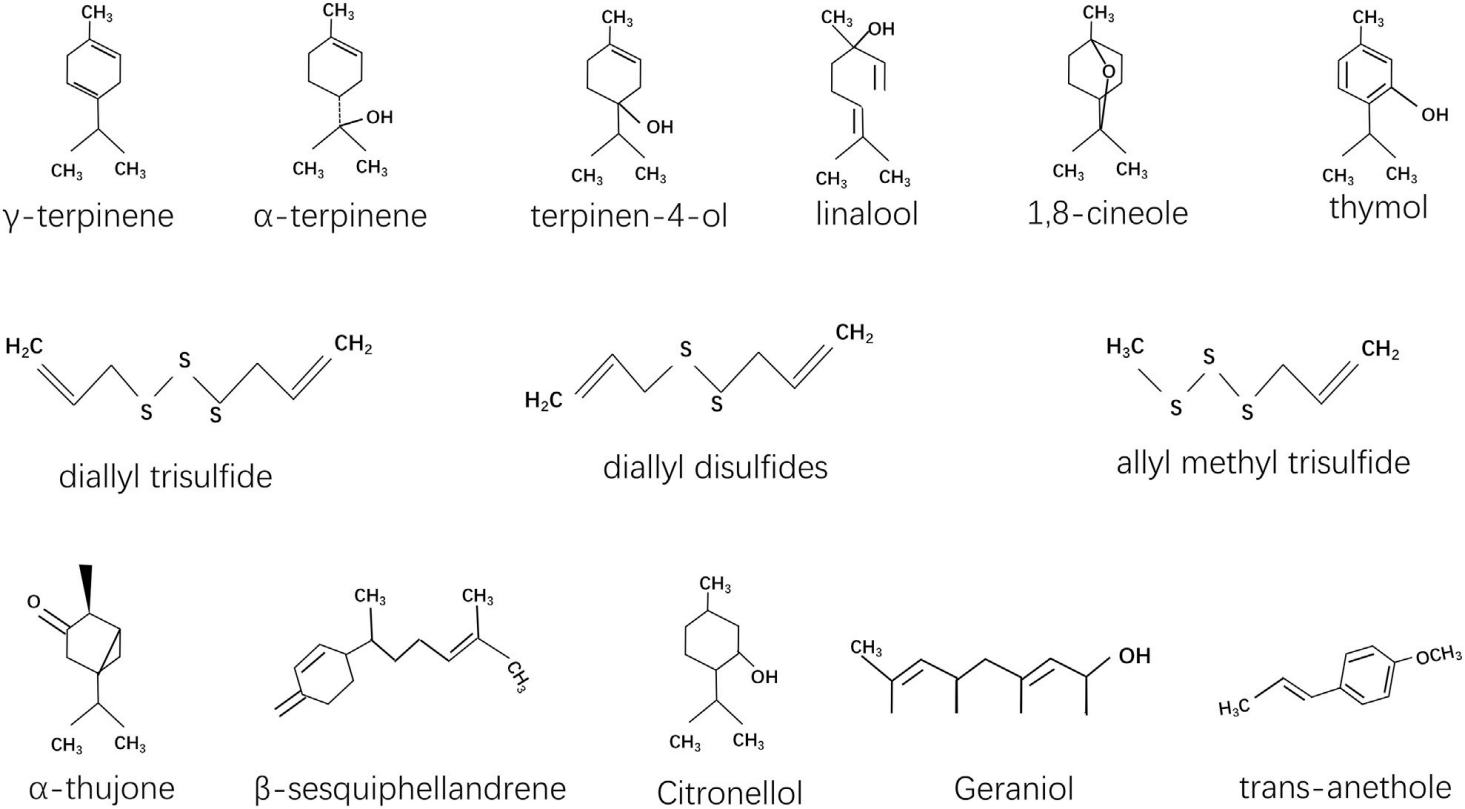
The structures of the main compositions which demonstrate the antioxidant potential in response to chemical overexposure.

An analysis of the structure-function relationship of these EOs may help to reveal how their distinct structural features contribute to their diverse biological activities. For example, the main compositions of EOs from plants (Table 4) that have shown potential in alleviating pesticide-induced hepatorenal damage include a variety of bioactive compounds. Notable constituents are γ-terpinene, α-terpinene, and terpinen-4-ol, which have potent antioxidant and anti-inflammatory properties (Kakouri et al., 2022). Sulfur compounds like diallyl trisulfide and diallyl disulfides provide protective effects, particularly against oxidative stress (Herrera-Calderon et al., 2021). Monoterpenes such as linalool, 1,8-cineole, and thymol offer anti-inflammatory and antimicrobial benefits, while α-thujone and β-sesquiphellandrene have notable hepatoprotective activity (Abou El-Soud et al., 2015; Fayoumi et al., 2022; Lombrea et al., 2020; Pokajewicz et al., 2021; Rehman et al., 2022). Other compounds like citronellol, geraniol, and trans-anethole are known for their detoxifying and antioxidant effects, collectively contributing to the mitigation of pesticide-induced hepatorenal damage (Galovičová et al., 2021; Kačániová et al., 2023; Sharopov et al., 2017). The structures of these main compositions are showed in Figure 2.

**TABLE 4:** The main compositions and contents of essential oils from plants that have shown potential in alleviating pesticide-induced hepatorenal damage.

Source	Main compositions	References
*Origanum majorana*	γ-terpinene (25.73%), α-terpinene (17.35%), terpinen-4-ol (17.24%)	Kakouri et al. (2022)
*Artemisia. campestris*	α-thujone (33.8%) and β-sesquiphellandrene (28.6%)	Rehman et al. (2022)
*Ocimum basilicum*	Linalool (48.4%), 1,8-cineol (12.2%), eugenol (6.6%), methyl cinnamate (6.2%), α-cubebene (5.7%), caryophyllene (2.5%), β-ocimene (2.1%) and α-farnesene (2.0%)	Abou El-Soud et al. (2015)
*Origanum vulgare*	Thymol (37.13%), gama-terpinene (9.67%), carvacrol (9.57%), carvacrol methyl ether (6.88), cis-alpha-bisabolene (6.80%), eucalyptol (3.82%), p-cymene (3.58%) and elemol (2.04%)	Lombrea et al. (2020)
*Lavandula stoechas*	Linalyl acetate (28.9%), linalool (24.3%), caryophyllene (7.9%), trans-3,7-dimethylocta-1,3,6-triene (4.6%), 4-terpineol (4.0%), lavandulyl acetate (3.5%), borneol (2.60%), and eucalyptol (2.05%)	Pokajewicz et al. (2021)
*Pelargonium hybrid*	Citronellol (30.2%), citronellyl formate (9.3%), geraniol (7.6%), isomenthone (4.1%), and linalool (3.2%)	Fayoumi et al. (2022)
Garlic (*Allium sativum* L.)	Diallyl trisulfide (44.21%), diallyl disulfides (22.08%), allyl methyl trisulfide (9.72%), 2-vinyl-4*H*-1,3-dithiine (4.78%), and α-bisabolol (3.32%)	Herrera-Calderon et al. (2021)
*Pelargonium graveolens*	β-citronello (29.7%), geraniol (14.6%), menthol (6.7%), and linalool (3.8%)	Kačániová et al. (2023)
*Thymus vulgari*	Thymol 48.1%, p-cymene 11.7%, 1,8-cineole 6.7%, γ-terpinene 6.1%, and carvacrol 5.5%	Galovičová et al. (2021)
*Foeniculum vulgare*	Trans-anethole (36.8%), α-ethyl-p-methoxy-benzyl alcohol (9.1%), p-anisaldehyde (7.7%), carvone (4.9%), 1-phenyl-penta-2,4-diyne (4.8%) and fenchyl butanoate (4.2%)	Sharopov et al. (2017)

## 7 Future perspectives

At optimal doses, EOs can exert maximal therapeutic benefits (Table 1–3). While most EOs exhibit dose-dependent effects, where their antioxidant and protective properties vary with concentration. Some research reports conflicting findings regarding their efficacy and safety. There is still a need for well-designed *in vivo* studies and human clinical trials to validate the efficacy and safety of EOs in preventing chemical-induced oxidative stress. Some essential oils, such as eucalyptus and camphor, have been reported to exhibit toxicity at high doses, causing adverse effects like neurotoxicity and hepatotoxicity (Lemmens-Gruber, 2020; Soni et al., 2023). One study focusing on acute, developmental, and reproductive toxicity using alternative *in vitro* and *in vivo* models suggested that certain EOs, including rosemary, citrus, and eucalyptus oils could exhibit dose-dependent toxicity, oxidative stress induction, and mucous membrane irritation (Lanzerstorfer et al., 2021). Nonetheless, studies have suggested that the toxicity concerns associated with EOs could be leveraged for their development as plant-based pesticides (Nonci et al., 2025). Therefore, while EOs show promise as natural detoxification agents by protecting the liver against oxidative stress, inflammation, and lipid metabolic disorders, careful consideration of their dosage is essential to maximize benefits while minimizing adverse effects.

There is limited research specifically on combining EOs with pharmaceutical antioxidants. However, the observed synergistic interactions among various natural compounds suggest potential benefits in such combinations. For example, mixtures containing oregano and thyme oils, as well as cranberry and rosemary extracts, have shown synergistic antioxidant effects, effectively extending the shelf life of meat products and improving sensory acceptance (Khodaei et al., 2023; Latoch et al., 2023). Given the promising results of natural compound combinations, further research is warranted to explore the synergistic potential of EOs with pharmaceutical antioxidants.

Clinical trials should assess the bioavailability, dosage, and long-term effects of EOs in humans, particularly in populations at high risk of chemical exposure. As chemical exposures continue to diversify with emerging industrial processes and pollutants, future research should expand the range of chemicals studied in relation to EO protective benefits. Investigating the efficacy of EOs against newer pollutants, such as microplastics, endocrine disruptors, and novel nanomaterials, will be essential for staying ahead of evolving environmental and occupational risks. Additionally, considering the inherent instability of EOs under environmental stresses such as temperature and light, the exploration of novel technologies to safeguard and enhance their characteristics and biological activities becomes imperative. To improve the bioavailability and stability of EO components, strategies such as microencapsulation have been explored (Ambrosio et al., 2020). This approach enhances oxidative stability, thermostability, photostability, shelf life, biological activity, and ensures targeted delivery of EOs. Finally, the sustainable production and harvesting of plants for essential oil extraction must be addressed. Establishing standardized protocols for EO composition and quality control is vital to ensure consistent therapeutic outcomes and minimize environmental impacts.

## 8 Conclusion

This review highlights the promising role of EOs as protective agents against oxidative stress induced by chemical overexposure. The evidence from studies on various chemicals-including pesticides, medications, nanoparticles, heavy metals, and organic compounds-demonstrates that EOs can mitigate oxidative damage through their antioxidant properties. Among them, EOs from *Origanum* species have shown notable efficacy in mitigating oxidative damage of pesticide overexposure. *Lavandula stoechas* EOs have exhibited protective properties against both pesticide-induced oxidative damage and overdoses of medications. Similarly, *Salvia officinalis L.* EOs have been identified for their potential to counteract the harmful effects of medication overdoses and organic chemical exposure. Furthermore, *Thymus vulgaris L.* EOs have demonstrated beneficial effects in mitigating oxidative damage resulting from medication overdoses and heavy metal exposure. The application of EOs in functional foods and pharmaceuticals presents significant potential for preventive and therapeutic use. However, further research is necessary to deepen our understanding of their mechanisms, optimize delivery systems, and confirm their efficacy in clinical settings. As chemical exposures continue to diversify in both environmental and occupational contexts, EOs represent a valuable avenue for future development in safeguarding human health. In conclusion, essential oils stand out as viable candidates for combating oxidative stress from chemical overexposure, paving the way for their integration into health-promoting interventions in both food and medicine.
